# Dataset meta-level and statistical features affect machine learning performance

**DOI:** 10.1038/s41598-024-51825-x

**Published:** 2024-01-19

**Authors:** Shahadat Uddin, Haohui Lu

**Affiliations:** School of Project Management, Faculty of Engineering, The University of Sydney, Forest Lodge, NSW 2037 Australia

**Keywords:** Applied mathematics, Computational science, Statistics

## Abstract

What dataset features affect machine learning (ML) performance has primarily been unknown in the current literature. This study examines the impact of tabular datasets' different meta-level and statistical features on the performance of various ML algorithms. The three meta-level features this study considered are the dataset size, the number of attributes and the ratio between the positive (class 1) and negative (class 0) class instances. It considered four statistical features for each dataset: mean, standard deviation, skewness and kurtosis. After applying the required scaling, this study averaged (uniform and weighted) each dataset’s different attributes to quantify its four statistical features. We analysed 200 open-access tabular datasets from the Kaggle (147) and UCI Machine Learning Repository (53) and developed ML classification models (through classification implementation and hyperparameter tuning) for each dataset. Then, this study developed multiple regression models to explore the impact of dataset features on ML performance. We found that kurtosis has a statistically significant negative effect on the accuracy of the three non-tree-based ML algorithms of the Support vector machine (SVM), Logistic regression (LR) and K-nearest neighbour (KNN) for their classical implementation with both uniform and weighted aggregations. This study observed similar findings in most cases for ML implementations through hyperparameter tuning, except for SVM with weighted aggregation. Meta-level and statistical features barely show any statistically significant impact on the accuracy of the two tree-based ML algorithms (Decision tree and Random forest), except for implementation through hyperparameter tuning for the weighted aggregation. When we excluded some datasets based on the imbalanced statistics and a significantly higher contribution of one attribute compared to others to the classification performance, we found a significant effect of the meta-level ratio feature and statistical mean and standard deviation features on SVM, LR and KNN accuracy in many cases. Our findings open a new research direction in understanding how dataset characteristics affect ML performance and will help researchers select appropriate ML algorithms for a possible optimal accuracy outcome.

## Introduction

Machine learning (ML) models have found applications across diverse fields, from healthcare and biomedical to finance and e-commerce^1^. Despite their widespread usage, the performance of ML models can vary based on the datasets to which they are applied. A crucial aspect of improving the performance metrics lies in understanding the intrinsic characteristics of datasets and how they interact with various methods. For instance, certain dataset features might bolster the accuracy of a specific technique, while other features could hinder the performance of others. Therefore, understanding these nuances can significantly enhance the predictability and reliability of ML models.

In this study, we delve into the relationship between specific dataset attributes, such as kurtosis, meta-level size, and ratio features, and the performance of ML models. Our primary aim is to uncover patterns that can guide researchers in selecting algorithms that align with the characteristics of their datasets. To achieve this, we conducted extensive experiments using five classification models: Support Vector Machine^2^, Decision Tree^3^, Random Forest^3^, Logistic Regression^4^ and K-Nearest Neighbour^5^. In ML literature, algorithms are often delineated into two primary categories: tree-based and non-tree-based. Tree-based algorithms, including Decision Trees and Random Forest, construct decision boundaries through hierarchical tree structures. Non-tree-based algorithms, such as Support Vector Machines and Logistic Regression, are underpinned by distinct foundational methodologies. Our research utilised 200 diverse UCI Machine Learning Repository and Kaggle tabular datasets. We analysed the variations in model performance concerning different meta-level and statistical features, focusing primarily on the accuracy performance metric. To ensure the robustness of our findings, we applied statistical tests to validate the observed performance differences across various scenarios.

The organisation of this manuscript is as follows: Section "Related works" summarises related work, and Section "Materials and methods" details our methodology, emphasising the datasets, dataset features, and machine learning models. Section "Results" presents the results, while Section "Discussion" comprehensively discusses our findings. Section "Conclusion" concludes the paper, synthesising our research's central insights and implications.

## Related works

ML methods have become popular and have been used extensively for addressing complex problems in different fields, such as healthcare^6^, commerce^7^, computer vision^8^ and natural language processing^9^. As ML applications have increased, there has also been a growing curiosity in contrasting their efficacy. This is reflected in the expanding body of research delving into the accuracy and effectiveness of various ML models in diverse scenarios.

The size of the dataset is a fundamental aspect that influences the performance of ML models^10^. Typically, a dataset with a larger sample size provides richer information, enabling the underlying ML model to capture detailed patterns, thereby enhancing its generalisation capabilities^11^. Usually, larger datasets improve classification outcomes, while smaller datasets often result in over-fitting^12,13^. In addition to the data size, Choi and Lee^14^ found that the subjectivity of the data affected ML performance for sentiment classification. They assigned a higher subjectivity score to data containing words such as 'best' or 'extremely' to express personal opinion and factual information.

On the other hand, the relationship between dataset size and ML performance is not always direct. Sun et al.^15^ observed that increasing the size might not yield substantial improvements in performance beyond a certain threshold, especially if the data is redundant or cluttered with noise. While researching object detection using non-parametric models, Zhu et al.^16^ observed that data quality and improved models are more important than the data size for better ML outcomes. Barbedo^17^ commented that using a limited dataset for training may bring many undesirable consequences, negatively impacting the model performance.

Class imbalance can skew performance measurements, leading to potential overestimation, prompting methods like over-sampling, under-sampling, and synthetic data generation as corrective measures^18^. Through an experiment on image data, Qu et al.^19^ noticed that class imbalance influenced ML performance. Class imbalance is defined when the frequency of one class in the underlying data is significantly higher than the other and vice versa. Many other studies in the literature also observed and explained how class imbalance affected ML performance. For example, Thabtah et al.^20^ pointed out that class imbalance is a common problem in behavioural science. They conducted extensive experiments using tenfold cross-validation on many datasets to study the impact of varying class imbalance ratios on classifier performance. In a review article, Ray^21^ provided inconsistent evidence regarding the effect of adding more features to an ML model on accuracy.

The inherent properties of the data are defined by its statistical attributes, such as mean, standard deviation, skewness and kurtosis. These attributes are pivotal in model performance, gauging data asymmetry and tail behaviour^22^. Recently, the variance ratio was introduced as an indicator of data variability^23^. These attributes can directly or indirectly influence the performance of ML models. For instance, data with a highly skewed distribution might require treatments or transformations suitable for non-normal distributions.

Most current studies explored the impact of meta-level dataset features (e.g., size, class imbalance ratio and number of attributes). To our knowledge, no study examined the effect of dataset statistical features on ML performance. In addition to three meta-level features (size, number of attributes and the ratio between positive and negative classes), this study considers four statistical features (mean, standard deviation, skewness and kurtosis). It will also explore how these seven dataset features affect the performance of five different ML algorithms.

## Materials and methods

### Data acquisition and preprocessing

This study explores how meta-level and statistical features of datasets affect ML performance. For this purpose, we considered 200 open-access tabular datasets from the Kaggle (147) and UCI Machine Learning Repository (53). Supplementary Table S1 details the source of these datasets. Each dataset addresses a binary classification problem, i.e., the targeted dependent variable can take one of the two possible values, either 0 or 1. Some sources have multiple datasets. For example, datasets D126–D174 are from the same Kaggle source, with the worldwide University ranking data from different ranking-producing organisations for various years. The same is true for datasets D175–D180, from the same UCI source for the Monk's problem. Kaggle provides powerful tools and resources for the data science and AI community, including over 256,000 open-access datasets^24^. The UCI Machine Learning Repository collects over 650 open-access datasets for the ML community to investigate empirically^25^.

The datasets used in this study have attributes or variables of a wide range. These attributes can take an extensive range of values across datasets. Some appeared on a Likert scale, which captures textual opinions in a meaningful order^26^. For example, a response could be good, very good or excellent against a question of how you feel. We first changed such responses into a chronological numerical order. Second, the responses of some categorical attributes do not make an expressive numerical order. An example of such an attribute is gender, which can be male or female. A binary transformation of this attribute would lead to a bias, especially for the statistical *mean* or *average* feature. For a dataset with more male responses, if we consider 1 for males, that dataset's mean or average feature will be larger and vice versa. Another example of such features is the marital status. For this reason, this study considers a target-based encoding to convert such categorical attributes to a quantitative score. The target-based encoding is an approach to replace a categorical variable using information from the dependent or target variable^27^. Third, the range for some attributes starts from a negative value. We shifted the range for those attributes so that its starting value is 0. If the range for an attribute is − 3 to 3, we move it from 0 to 6 by adding 3 to all instances. This study's final preprocessing is to convert all attributes for each dataset into a range of 0 and 1 by following the min–max scaling approach^28^, which ensures their uniform value range across datasets. A dataset's attribute (e.g., duration) can be 1–10 h. The value for another variable (e.g., age) of the same or different dataset may range between 18 and 120 years. We divide each instance of an attribute by the magnitude of the range of that attribute. For example, if the adult age range is 18–120 years of an attribute, we divide each instance by 102 (120–18). Such a normalisation also neutralises the impact of considering different units for the same attribute. Age could be in year or month, but the relative difference across instances will remain the same with this normalisation approach. The conversion of each attribute into a range of 0–1 ensures the quantification of each statistical feature is from the same value range across datasets.

### Dataset features

This study considers three meta-level and four statistical features to investigate their influence on the performance of five classical supervised ML algorithms. We quantify these seven features and accuracy values against various ML algorithms for each dataset.

#### Meta-level features

For each dataset, this study considers three meta-level features. They are the number of attributes or variables used to classify the target variable, dataset size and the ratio between the number of yes (or positive) and no (negative) classes. A very high or low ratio value results in a class imbalance issue^29^. Hence, including this meta-level feature will help explore how the presence of class imbalance affects ML performance. The dataset size, or simply size, is the number of instances of that dataset. If a dataset consists of 100 cases with 60 positive and 40 negative samples, the ratio will be 1.25 (60 ÷ 40).

#### Statistical features

The four statistical features considered in this study for each dataset are mean or average, standard deviation, skewness and kurtosis.

##### Mean or average

For a set of $N$ numbers $({X}_{1},{X}_{2}\dots {X}_{N})$, the following formula can quantify the mean or average ($\overline{X }$) value.

$$\overline{X }=\frac{\sum_{i=1}^{N}{X}_{i}}{N}$$

##### Standard deviation

**Figure 1:**
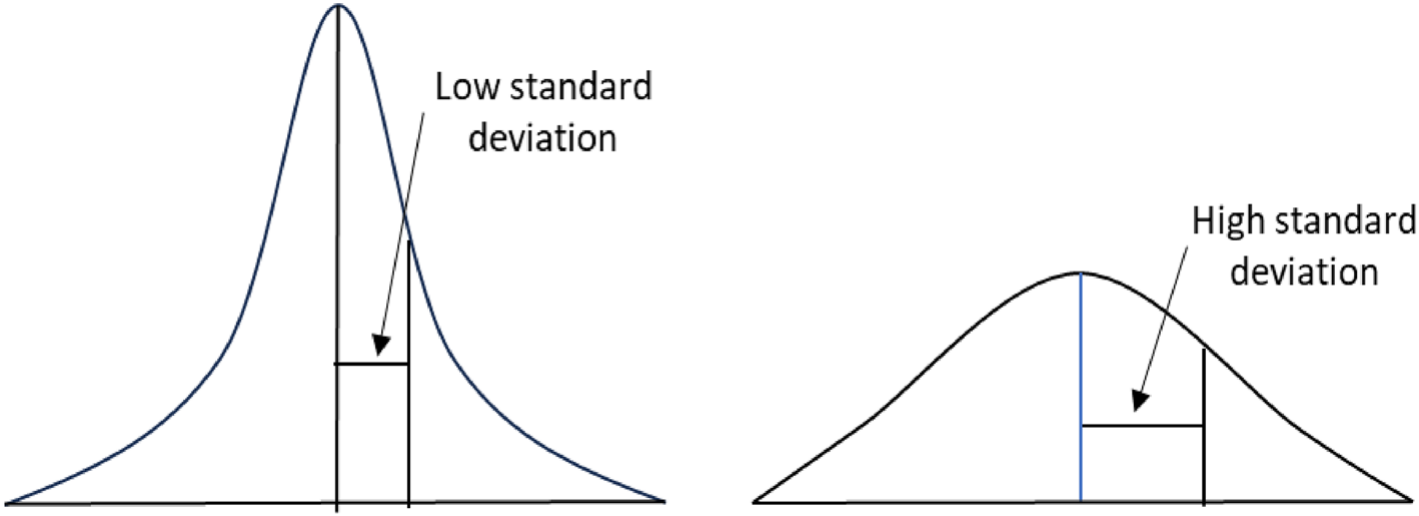
Illustration of low and high standard deviation.

Standard deviation is a commonly used descriptive statistical measure that indicates how dispersed the data points are concerning the mean^30^. It summarises the difference of each data point from the mean value (Fig. 1). A low standard deviation of a given data demonstrates that its data points are clustered tightly around its mean value. Conversely, a high standard deviation indicates that data points are spread out over a broader range. For a dataset with size $N ({X}_{1},{X}_{2}\dots {X}_{N})$ and mean $\overline{X }$, the formula for the standard deviation (SD) is as follows.

$$SD=\sqrt{\frac{\sum_{i=1}^{N}{\left({X}_{i}-\overline{X }\right)}^{2}}{N-1}}$$

##### Skewness

**Figure 2:**
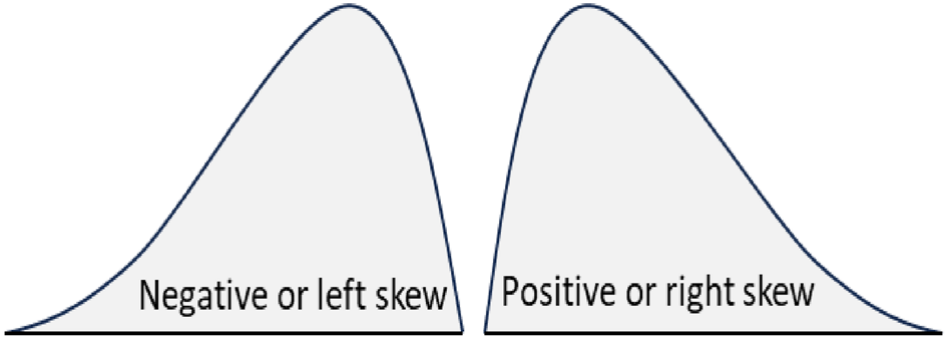
Illustration of left and right skewed distribution.

Skewness measures how far a particular distribution deviates from a symmetrical normal distribution^30^. The skewness value can be positive, zero or negative (Fig. 2). The left tail is longer for negatively skewed data. It is the right tail, which is longer for positively skewed data. Both tails are symmetrical for unskewed data. The following formula can measure the skewness (${\widetilde{\mu }}_{3}$) of a given data $({X}_{1},{X}_{2}\dots {X}_{N})$.

$${\widetilde{\mu }}_{3}=\frac{\sum_{i=1}^{N}{({X}_{i}-\overline{X })}^{3}}{(N-1)\times {SD}^{3}}$$

where, $\overline{X }$ and $SD$ are the mean and standard deviation of the data, respectively. In the above formula, if the value of ${\widetilde{\mu }}_{3}$ is greater than 1, the distribution is right-skewed. It is left-skewed for ${\widetilde{\mu }}_{3}$ is less than -1. The tail region may be the source of outliers for skewed data, which could adversely affect the performance of any statistical models based on that skewed data. Models that assume the normal distribution of the underlying data tend to perform poorly with highly skewed (positive or negative) data^30^.

##### Kurtosis

For the probability distribution of a real-valued random variable, kurtosis quantifies the level of existing tailedness within that distribution^30^. It can identify whether the data are heavy-tailed or light-tailed relative to the normal distribution. Here is the formula to quantify the kurtosis (${\beta }_{2}$) of a dataset $({X}_{1},{X}_{2}\dots {X}_{N})$ with mean and standard deviation of $\overline{X }$ and $SD$, respectively.

$${\beta }_{2}=\left\{\frac{N(N+1)}{(N-1)(N-2)(N-3)}\sum_{i=1}^{N}{\left(\frac{{X}_{i}-\overline{X}}{SD }\right)}^{4}\right\}-\frac{3{(N-1)}^{2}}{(N-2)(N-3)}$$

**Figure 3:**
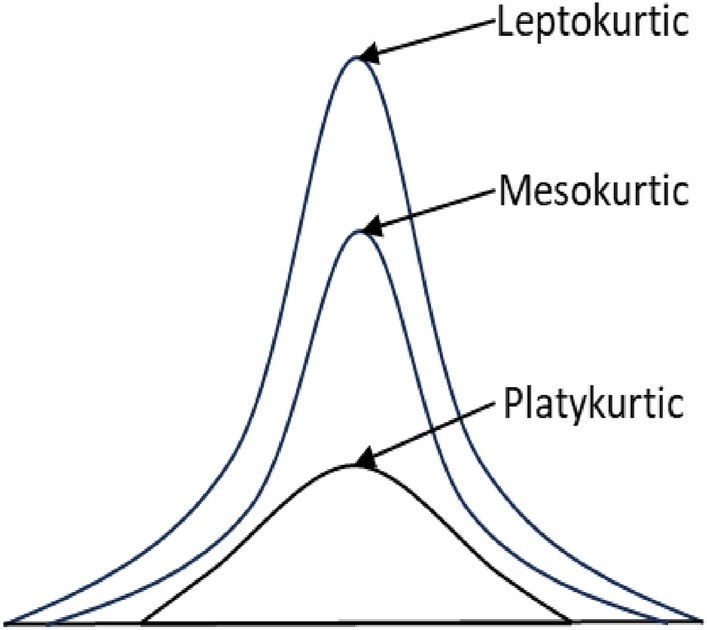
Illustration of leptokurtic, mesokurtic and platykurtic distribution.

Based on the kurtosis value (${\beta }_{2}$), a distribution can be leptokurtic, mesokurtic and platykurtic (Fig. 3). A standard normal distribution has a kurtosis value of 3, known as mesokurtic. An increased kurtosis (> 3), known as leptokurtic, makes the peak higher than the normal distribution. A decreased kurtosis (< 3), known as platykurtic, corresponds to a broadening of the peak and thickening of the tails. Excess kurtosis indicates the presence of many outliers presented in the dataset, which could negatively impact the performance of any statistical models based on that dataset^30^.

#### Feature value quantification

The online open-access source for each dataset contains information on the number of attributes and instances (dataset size) with further details on the positive and negative splits. The third meta-level ratio feature has been calculated by dividing the number of positive cases by the number of negative instances. We followed the same approach to quantify each of the four statistical features for a given dataset. First, we calculated the underlying feature value for each dataset attribute. If the underlying feature is the skewness and the given dataset has six attributes, we then calculate the skewness of each attribute. After that, we aggregate these six skewness values by taking their average value. We also follow a weighted approach to aggregate them using each attribute's principal component analysis (PCA) score as its weight. PCA is a popular dimensionality reduction technique that can assign scores to each feature based on their ability to explain the variance of the underlying dataset^31^.

### Machine learning algorithms and experimental setup

This study considers five supervised ML algorithms to investigate how dataset features affect their performance. Two (Random forest and Decision tree) are tree-based, and the remaining three (Support vector machine, Logistic Regression and K-nearest neighbours) do not use any tree structure for the classification task.

Decision Trees (DT) are non-parametric methods partitioning datasets into subsets based on attribute values, though they can sometimes overfit^3^. Random Forest (RF) is an ensemble learning method that constructs multiple decision trees during training and outputs the mode of the classes for classification or the mean prediction for regression tasks, thereby reducing the overfitting risk associated with individual decision trees^32^. Support Vector Machine (SVM) classifies data by determining the best hyperplane that divides a dataset into classes^2^. Logistic Regression (LR) analyses datasets where independent variables determine a categorical outcome, commonly used for binary classification^33^. Lastly, the K-Nearest Neighbours (KNN) algorithm classifies an input based on the most common class among its nearest *K* examples in the training set. It offers versatility at the cost of computational intensity^34^.

This study used the Scikit-learn library^35^ to implement the five ML algorithms with the 200 open-access tabular datasets considered in this study. Each dataset underwent an 80:20 split for the training and test data separation. We followed a five-fold cross-validation for training model development. For other experimental setups, this study used the default settings of the Scikit-learn library. Moreover, this study considered accuracy as the performance measure for ML algorithms. It represents the percentage of correct classifications made by the underlying ML model. In addition to the basic implementation using the Scikit-learn library, this study implemented all ML algorithms through hyperparameter tuning of different relevant parameters. Hyperparameter tuning is selecting a set of optimal parameters for the ML algorithm to boost its model performance^36^. We used the GridSearchCV function from Scikit-learn to tune different hyperparameters for different ML algorithms, such as the kernel type and C value for SVM and the *k* value in KNN.

After quantifying three meta-level and four statistical features and the ML accuracy for each of the 200 tabular datasets, this study applied multiple linear regression to explore their impact on ML performance. We used IBM SPSS Statistics software version 28.0.0.0^37^ for multiple linear regression modelling.

## Results

This study follows a data-driven approach to explore the impact of dataset meta-level and statistical features on the performance of five ML algorithms by using 200 open-access tabular datasets. Table 1 presents the basic statistics of the 200 open-access tabular datasets used in this study. Most (147 out of 200) are from the Kaggle^24^. Only 53 datasets are from the UCI Machine Learning Repository^25^. 168 (84%) datasets are from five primary contexts: Disease, University ranking, Sports, Fiance and Academia.

**Table 1:** Basic statistics of 200 open-access tabular datasets used in this study.

Item	Range	Value (%)
Total datasets		200
Attribute	[2–2548]	
Instance	[19–319,795]	
Dataset contexts (top five)		
Disease		66(33%)
University ranking		50 (25%)
Sports		23 (11.5%)
Finance		15 (7.5%)
Academia		14 (7%)
Dataset source		
Kaggle		147 (735%)
UCI machine learning repository		53 (26.5%)

We used two approaches to implement the five ML algorithms considered in this study over the 200 open-access tabular datasets: classic implementation and hyperparameter tuning. On the other hand, this study used two techniques in aggregating the statistical attributes: the uniform approach and the weighted approach based on PCA. Such considerations of different implementation approaches and aggregating techniques lead to four versions of the dataset consisting of seven meta-level and statistical features as independent variables and the performance of different ML algorithms as dependent variables. Moreover, we considered two other versions of the dataset instance created with classic ML implementation and uniform aggregation: excluding entries for extremely imbalanced individual datasets and excluding individual datasets having a single feature with a high PCA value. Therefore, we need to develop six multiple regression models to investigate the impact of dataset meta-level and statistical features on machine learning performance. This paper presents the findings in the following two subsections. The first subsection details the results for all four dataset variants with the classic ML implementation: (i) classic ML implementation with uniform aggregation of four statistical features, (ii) classic ML implementation with weighted aggregation, (iii) exclude entries for highly imbalanced datasets from the first variant, and (iv) exclude entries for datasets having a single feature with a very high PCA value from the first variant. The second subsection summarises two multiple regression results from the implementations through the hyperparameter tuning: one with uniform aggregation and the other with the weighted aggregation of statistical features.

### Classic ML implementation

This subsection details the results for four dataset variants that underwent the classic ML implementation. Table 2 presents the results of multiple regression models that explored the impact of three meta-level and four statistical dataset features on ML algorithm performance for the dataset variant with classic ML implementation and uniform aggregation for statistical features. We followed a five-fold cross-validation approach to implementing these ML algorithms to the training data. The statistical kurtosis feature negatively impacted the accuracy of all non-tree-based ML algorithms (i.e., SVM, LR and KNN) at *p* ≤ 0.05 level. The meta-level ratio feature revealed a statistically positive impact on these three ML algorithms, either at p ≤ 0.05 (KNN) or at *p* ≤ 0.10 (SVM and LR) levels. In addition, the statistical measures of mean and skewness positively impacted both SVM and KNN accuracy at different statistically significant levels (p ≤ 0.05 or p ≤ 0.10). Notably, the two tree-based ML algorithms (DT and RF) do not display any statistically significant association with the seven dataset features considered in this study.

**Table 2:** Multiple regression results for the accuracy of five machine learning algorithms (classic implementation with uniform aggregation for four statistical features).

	Standardised beta	t-value	Significance
(a) The accuracy of the support vector machine (*SVM_accuracy*) is the dependent variable ($${R}^{2}=0.09)$$			
Meta features			
No. of attributes	0.080	1.155	0.250
Size	0.012	0.164	0.870
Ratio	0.136	1.928	0.055
Statistical features			
Mean	0.242	1.939	0.054
Standard deviation	0.025	0.327	0.744
Skewness	0.496	1.778	0.077
Kurtosis	− 0.631	− 2.585	0.010
(b) The accuracy of the decision tree (*DT_accuracy*) is the dependent variable ($${R}^{2}=0.01)$$			
Meta features			
No. of attributes	0.026	0.365	0.716
Size	0.050	0.671	0.503
Ratio	0.049	0.667	0.506
Statistical features			
Mean	0.003	0.023	0.982
Standard deviation	0.069	0.852	0.395
Skewness	0.073	0.250	0.803
Kurtosis	− 0.046	− 0.179	0.858
(c) The accuracy of the random forest (*RF_accuracy*) is the dependent variable ($${R}^{2}=0.01)$$			
Meta features			
No. of attributes	0.026	0.366	0.715
Size	0.050	0.671	0.503
Ratio	0.049	0.667	0.506
Statistical features			
Mean	0.003	0.022	0.982
Standard deviation	0.069	0.850	0.396
Skewness	0.072	0.249	0.803
Kurtosis	− 0.045	− 0.178	0.859
(d) The accuracy of the logistic regression (*LR_accuracy*) is the dependent variable ($${R}^{2}=0.08)$$			
Meta features			
No. of attributes	0.093	1.333	0.184
Size	− 0.067	− 0.928	0.355
Ratio	0.131	1.839	0.068
Statistical features			
Mean	0.201	1.605	0.110
Standard deviation	− 0.086	− 1.108	0.269
Skewness	0.401	1.432	0.154
Kurtosis	− 0.511	− 2.081	0.039
(e) The accuracy of the K-nearest neighbour (*KNN_accuracy*) is the dependent variable ($${R}^{2}=0.09)$$			
Meta features			
No. of attributes	0.041	0.593	0.554
Size	0.106	1.474	0.142
Ratio	0.140	1.982	0.049
Statistical features			
Mean	0.275	2.208	0.028
Standard deviation	− 0.115	− 1.488	0.138
Skewness	0.528	1.897	0.059
Kurtosis	− 0.640	− 2.623	0.009

Predictor dataset features are categorised into meta and statistical groups.

After excluding 27 highly imbalanced datasets, we developed multiple regression models for the five algorithms created with classic ML implementation and uniform aggregation. Although there is no exact definition of an imbalanced dataset^18^, this study evaluated a dataset as extremely imbalanced if one of its classes has a frequency of ≥ 90% or ≤ 10%. Table 3 shows the corresponding multiple regression results for the five ML algorithms in a summarised format, excluding the underlying models' *beta* and *t-test* details. We noticed two significant findings from this imbalanced-free regression results. First, mean and kurtosis features impacted the accuracy of three non-tree-based ML algorithms (SVM, LR and KNN) at p ≤ 0.05 or p ≤ 0.10. This impact is in a positive direction for the mean but a negative direction for kurtosis. Second, none of the seven dataset features has any statistically significant effect on the accuracy of two tree-based ML algorithms (DT and RF). Further, the meta-level size feature negatively affected SVM and LR accuracy at *p* ≤ 0.05 and *p* ≤ 0.10, respectively. Skewness revealed a similar significant effect (*p* ≤ 0.05) for the SVM and KNN accuracy but in the opposite direction.

**Table 3:** Summarised multiple regression results (classic machine learning implementation with uniform aggregation) excluding extremely imbalanced 27 datasets.

	SVM	DT	RF	LR	KNN
Meta features					
No. of attributes	–	–	–	–	–
Size	(− ve)**	–	–	(− ve)*	–
Ratio	–	–	–	–	–
Statistical features					
Mean	(+ ve)**	–	–	(+ ve)**	(+ ve)**
Standard deviation	–	–	–	–	(− ve)*
Skewness	(+ ve)**	–	–	–	(+ ve)**
Kurtosis	(− ve)**	–	–	(− ve)*	(− ve)**

A double asterisk (**) and a single asterisk (*) indicate that the underlying impact is significant at ≤ 0.05 and ≤ 0.10 levels, respectively. A (+ ve) or (− ve) shows the beta-value sign for the corresponding feature variable. A hyphen (–) indicates no statistically significant effect.

While implementing ML algorithms against the datasets of this study, we noticed that a single attribute significantly impacted the underlying ML classification performance for a few datasets. We found 35 datasets where a single variable or attribute revealed ≥ 20% variance explained according to PCA^31^ of the Scikit-learn. We removed these datasets and then applied multiple regressions on the remaining 165 (200–35) datasets. Table 4 presents the corresponding results. Mean and kurtosis revealed the same impact on all ML algorithms, as in Table 3. These two features showed a statistically significant effect on SVM, LR and KNN accuracy at *p* ≤ 0.05 and did not impact the performance of the two tree-based ML algorithms (DT and RF). The meta-level ratio feature positively affected the performance of the three non-tree-based ML algorithms (SVM, LR and KNN) at *p* ≤ 0.10. Standard deviation negatively affected LR and KNN accuracy at *p* ≤ 0.05. Skewness positively affected SVN and KNN accuracy at *p* ≤ 0.10 and *p* ≤ 0.05, respectively.

**Table 4:** Summarised results for five multiple regression models (classic machine learning implementation with uniform aggregation) excluding 35 datasets where a single attribute significantly impacted the classification performance (i.e., variance explained is ≥ 20%).

	SVM	DT	RF	LR	KNN
Meta features					
No. of attributes	–	–	–	–	–
Size	–	–	–	–	–
Ratio	(+ ve)*	–	–	(+ ve)*	(+ ve)*
Statistical features					
Mean	(+ ve)**	–	–	(+ ve)**	(+ ve)**
Standard deviation	–	–	–	(− ve)**	(− ve)**
Skewness	(+ ve)*	–	–	–	(+ ve)**
Kurtosis	(− ve)**	–	–	(− ve)**	(− ve)**

A double asterisk (**) and a single asterisk (*) indicate that the underlying impact is significant at ≤ 0.05 and ≤ 0.10 levels, respectively. A (+ ve) or (− ve) shows the beta value sign for the corresponding feature variable. A hyphen (–) indicates no statistically significant effect.

Table 5 summarises the multiple regression results for the dataset variant created with classic ML implementation and weighted aggregation of four statistical measures. Ratio and kurtosis significantly impact non-tree-based SVM, LR and KNN approaches. Statistical mean and standard deviation measures revealed statistically significant positive and negative effects on LR and KNN, respectively, at *p* ≤ 0.05. Interestingly, the meta-level number of attributes discloses a significant positive impact with tree-based DT and RF algorithms at *p* ≤ 0.05.

**Table 5:** Summarised multiple regression results for the accuracy of five machine learning algorithms (classic implementation with weighted aggregation for four statistical features).

	SVM	DT	RF	LR	KNN
Meta features					
No. of attributes	–	(+ ve)**	(+ ve)**	–	–
Size	–	–	–	–	–
Ratio	(+ ve)*	–	–	(+ ve)**	(+ ve)*
Statistical features					
Mean	–	–	–	(+ ve)**	(+ ve)**
Standard deviation	–	–	–	(− ve)**	(− ve)**
Skewness	–	–	–	–	(+ ve)*
Kurtosis	(− ve)*	–	–	(− ve)**	(− ve)**

A double asterisk (**) and a single asterisk (*) indicate that the underlying impact is significant at ≤ 0.05 and ≤ 0.10 levels, respectively. A (+ ve) or (− ve) shows the beta value sign for the corresponding feature variable. A hyphen (–) indicates no statistically significant effect.

Comparing the findings from Tables 2, 3, 4, 5, it is evident that the statistical feature of *kurtosis* always showed a statistically significant negative effect on the performance of the three non-tree-based ML algorithms (SVM, LR and KNN). None of the four statistical features significantly impacted the performance of the two tree-based ML algorithms (DT and RF). The three meta-level features also revealed the same impact except for the classic implementation with weighted aggregation (Table 5), where the number of attributes showed a statistically positive effect on DT and RF performance at *p* ≤ 0.05. The mean feature showed a statistically significant positive impact in all cases except LR for the original research data for classic implementation with uniform aggregation of statistical measures. The meta-level ratio feature also divulged a statistically significant positive effect on SVM, LR and KNN performance for dataset variants except for the trimmed version that excludes highly imbalanced 27 datasets (Table 3).

### ML implementation through hyperparameter tuning

Table 6 reports the multiple regression results for the accuracy measure of the five ML algorithms implemented through hyperparameter tuning. We followed uniform and weighted aggregation approaches to quantify the four statistical features for each dataset. Kurtosis showed a statistically significant impact for most cases except SVM implementation with the weighted aggregation. The meta-level ratio feature showed a substantial effect with LR and KNN with both aggregation approaches. DT and RF did not significantly relate to any of the seven features for uniform aggregation. However, they revealed a significant statistical relation with the number of attributes for the weighted aggregation of statistical features. Notably, SVM did not significantly correlate with the seven features for the weighted aggregation.

**Table 6:** Summarised multiple regression results for the accuracy of five machine learning algorithms (implementation through hyperparameter tuning with uniform and weighted aggregation for four statistical features).

	Uniform aggregation					Weighted aggregation				
	SVM	DT	RF	LR	KNN	SVM	DT	RF	LR	KNN
Meta features										
No. of attributes	–	–	–	–	–	–	(+ ve)*	(+ ve)**		–
Size	–	–	–	–	–	–	–	–		–
Ratio	(+ ve)*	–	–	(+ ve)**	(+ ve)*	–	–	–	(+ ve)**	(+ ve)*
Statistical features							–		(+ ve)*	
Mean	(+ ve)*	–	–	–	(+ ve)**	–	–	–	(− ve)*	(+ ve)**
Standard deviation	(+ ve)*	–	–	–	–	–	–	–	–	(− ve)**
Skewness	(+ ve)*	–	–	–	(+ ve)*	–	–	–	–	–
Kurtosis	(− ve)**	–	–	(− ve)*	(− ve)**	–	–	–	(− ve)*	(− ve)**

A double asterisk (**) and a single asterisk (*) indicate that the underlying impact is significant at ≤ 0.05 and ≤ 0.10 levels, respectively. A (+ ve) or (− ve) shows the beta value sign for the corresponding feature variable. A hyphen (–) indicates no statistically significant effect.

## Discussion

In most cases, kurtosis showed a statistically negative effect on the three non-tree-based ML algorithms except for SVM with hyperparameter tuning and weighted aggregation. A dataset with a higher kurtosis value (leptokurtic) offers lower SVM, LR and KNN accuracy values, and vice versa. Such leptokurtic datasets have a higher pick and tend to have heavier tails on both sides than the standard normal distribution, making them inclined to extreme outlier values. Outliers are the data points located far away from other data points and the distribution. The SVM decision boundary considers a set of points located on the hyperplanes on both sides. The position of this boundary line depends on the distance between points on the opposite hyperplanes. Throughout this process, SVM effectively ignores data points far from the decision boundary, potentially making it vulnerable to outliers^38^. A basic assumption of LR is that independent variables have a linear relation with the dependent variable. This assumption can be compromised by outliers in the input data^30^. Since it considers all data points for classification, KNN performance is highly susceptible to outliers^39^.

DT and RF do not reveal a statistically significant relation with any of the four statistical features considered in this study. For classification, these two tree-based ML algorithms do not use distance measures and consider no linearity assumption between independent and dependent attributes. For this reason, they can effectively handle non-linear data for classification tasks. DT is a decision-support hierarchical tree-like model based on conditional control statements. RF is an ensemble learning method consisting of several DTs. The functional approach to conducting classification and not relying on the linearity assumption may make DT and RF not sensitive to any meta-level and statistical measures considered in this study. Further research is needed to reach a more concrete conclusion in this regard.

Like the current literature, such as^14–16^, the meta-level size and ratio features show inconsistent effects on the accuracy of the three non-tree-based ML algorithms for different datasets. Size has been found to negatively impact SVM and LR performance for datasets that are not highly imbalanced. This relation was insignificant for KNN for the same set of datasets. This relation was also not significant for the other dataset variants. A similar inconsistent relationship has been noticed for ratio and the number of attribute measures with the three non-tree-based ML algorithms. Although few statistical features showed a more consistent effect on these three non-tree-based ML algorithms, the way they contribute to the ML training process remains largely unknown, primarily due to the complex learning nature of ML algorithms. Future research could address this issue further in depth.

Our findings could help potential researchers select appropriate non-tree-based ML algorithms for classification modelling based on the features of the underlying research dataset. For example, for a dataset with a high negative kurtosis score, all three non-tree-based ML algorithms would be better for optimal classification outcomes. These three algorithms should also be desirable for datasets with higher positive instances than their counterparts. KNN would result in a better classification outcome among these three ML algorithms for a balanced dataset with a negative standard deviation.

This study used the min–max scaling for data normalisation. This approach allows each dataset feature to have a scale between 0 and 1. Other normalising strategies exist, such as log scaling and z-score. However, they are not suitable for this research. We cannot consider the z-score approach since this study evaluated mean and standard deviation as the statistical features. Therefore, if we apply the z-score normalisation approach, the values of these two features will be 0 and 1, respectively, of these two features for each dataset. On the other hand, the highest value of some features is extensive in some datasets. For example, the first dataset^40^ has three variables (blood pressure, cholesterol and max heart rate), with the highest value of ≥ 200. Considering a log scaling to this dataset will make a statistical bias compared to another dataset that does not have variables with such high scores.

## Conclusion

Non-tree-based ML algorithms are sensitive to dataset features. We found a statistically significant effect of kurtosis on the three non-tree-based ML algorithms across all three versions of the research data. Meta-level ratio and statistical mean features often significantly impact these three ML algorithms. Conversely, tree-based ML algorithms are not sensitive to any of the seven measures considered in this study. Future studies can explore and reveal this difference in the effects of dataset features on performance between non-tree-based and tree-based ML algorithms. Until then, based on the seven features of a given dataset, this research could provide helpful insight into the selection of ML algorithms and their expected accuracy outcomes.

This study concentrates on five supervised ML algorithms and the binary classification of their implementation on tabular datasets. The target variable of the 200 datasets we used has only two categories. A possible extension of this study is to consider other tabular datasets having more than two classes. Another possible extension of this study is to evaluate other ML algorithms based on tabular data, such as the ensemble approaches of boosting and stacking. A third possible extension would be the consideration of deep learning methods^41^. In addition to these potential future research opportunities, our findings will open a new arena in understanding how dataset meta-level and statistical features impact ML performance.

### Supplementary Information

Supplementary Table 1.

## Data Availability

The 200 datasets used in this study are publicly available from open-source repositories.
